# Collision of Basal Cell Carcinoma and Sebaceous Carcinoma Extirpated With Mohs Micrographic Surgery

**DOI:** 10.7759/cureus.18015

**Published:** 2021-09-16

**Authors:** Austin B Ambur, Timothy Nyckowski, Etan Marks, Joshua Spanogle

**Affiliations:** 1 Dermatology, Kansas City University-Graduate Medical Education Consortium/Advanced Dermatology and Cosmetic Surgery, Oviedo, USA; 2 Dermatopathology, Kansas City University-Graduate Medical Education Consortium/Advanced Dermatology and Cosmetic Surgery, Oviedo, USA

**Keywords:** dermatologic surgery, mohs, neoplasm, collision, sebaceous carcinoma, basal cell carcinoma

## Abstract

Collision tumors are neoplasms composed of two or more distinct cellular lineages coexisting at the same anatomic site. Incomplete biopsy, partial pathological slide examination or failure to include this diagnosis into the clinical differential may complicate and delay appropriate therapy. Although collision tumors are well documented, basal cell carcinoma (BCC) occurring with sebaceous carcinoma (SC) has only been reported in a single case report. The aim of the authors is to present a case of collision BCC and SC to highlight a rare clinicopathological case. We also present this case to advise caution to detect mimickers of BCC that warrant greater clinical workup and use this case to emphasize the importance of Mohs micrographic surgery for the treatment of SC.

## Introduction

Basal cell carcinoma (BCC) is the most common cancer worldwide, while sebaceous carcinoma (SC) is a rare neoplasm that develops from sebaceous glands. SC most commonly presents on the face, particularly the periocular region, and is often misdiagnosed as a benign entity such as a chalazion or a malignant lesion such as BCC and SCC. Collision tumors are neoplasms composed of two or more distinct cellular lineages coexisting at the same anatomic site. Incomplete biopsy, partial pathological slide examination or failure to include this diagnosis into the clinical differential may complicate and delay appropriate therapy. We present the second reported case of collision BCC and SC.

## Case presentation

**Figure 1:**
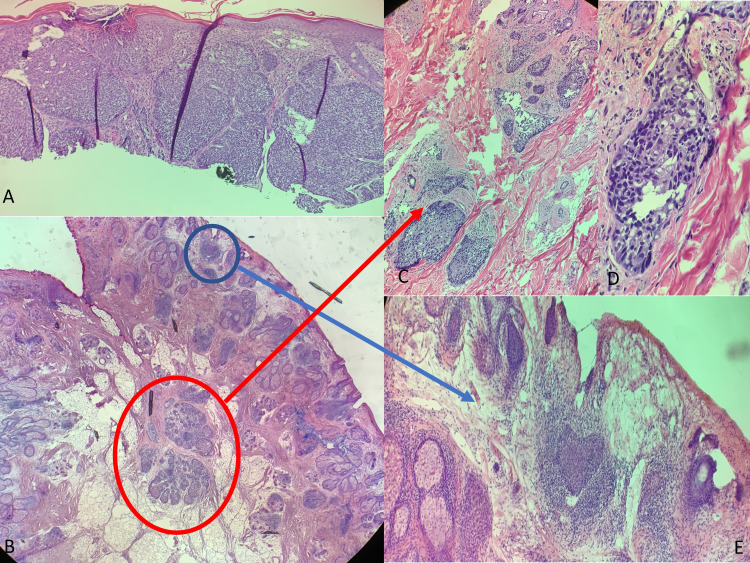
A) Hematoxylin and eosin (H&E) stain 10x original biopsy showing basal cell carcinoma. B) 2x Mohs specimen showing deep focus of sebaceous carcinoma and superficial remnant of basal cell carcinoma. C,D) 20x and 40x of sebaceous carcinoma. E) 10x of residual basal cell carcinoma.

A 76-year-old male with a history of BCC and squamous cell carcinoma presented to clinic for Mohs micrographic surgery to extirpate a biopsy-proven BCC, two weeks after initial biopsy (Figure 1A). Mohs surgery was performed with frozen sections, and the first stage revealed atypical basaloid cells surrounding atypical foamy cells with scalloped nuclei at the deep margin, concerning for SC (Figures 1B-1D). The atypical cells were cleared on the second stage, leaving a 2.2 x 1.4 cm surgical defect size. Though SC was favored, given the equivocal pathology on frozen section (BCC with adnexal differentiation vs SC), these slides were sent to dermatopathology for tumor confirmation. A dermatopathologist re-examined the initial biopsy slide, and confirmed nodular BCC alone to be present, as seen in Figure 1A, with morphologic features being distinct and different from the sebaceous carcinoma (Figures 1B-1D). The first Mohs stage was re-embedded for permanent sectioning and immunohistochemical staining. SC at the deep margin was confirmed as seen in Figures 1B-1D, as well the presence of a separate focus of BCC extending into the superficial dermis, as seen in Figure 1B and Figure 1E. The dermatopathologist agreed with the diagnosis of sebaceous carcinoma and favored this to be a collision lesion rather than a contiguous BCC with sebaceous differentiation. The patient continues to follow up at six-month intervals for tumor surveillance. 

## Discussion

BCC arises from keratinocyte progenitor cells of the interfollicular epidermis and upper infundibulum that undergo neoplastic transformation [1]. Standard excision with a 4-mm margin is effective for most subtypes of BCC, with very rare incidence of metastatic spread.

SC is a rare malignant adnexal neoplasm that can develop in any region where sebaceous glands are present. SC are typically highly infiltrative neoplasms that display pagetoid spread into the epidermis and lacks peripherial palisading and retraction clefts as seen in BCC [2]. Characteristic histology shows irregular lobular pattern with variable sebaceous differentiation [1]. Undifferentiated cells appear as foamy cells with scalloped nuclei. The histological diagnosis can be challenging because it can mimic several other lesions, including BCC [2]. The prognosis of SC is variable, with metastatic rates around 8% and some tumors having multifocal or discontinuous spread [3]. Most SC arise sporadically, however 43% are associated with Muir-Torre syndrome (MTS) [4]. MTS is an inherited condition characterized by a predisposition to sebaceous neoplasms and visceral malignancies. Sebaceous adenoma is the most common sebaceous neoplasm followed by sebaceous epithelioma and SC [1]. Colorectal adenocarcinoma is the most common visceral malignancy associated with MTS, as well as endometrial, ovarian, small bowel, pancreatic, hepatobiliary tract, brain, upper uroepithelial tract, breast, and lung [1]. It is recommended to evaluate for possible MTS after diagnosing SC, with a thorough history and physical examination including palpation of lymph nodes. Positive responses to personal history, family history, or internal malignancies that characterize MTS should prompt appropriate workup, including colonoscopy [4].

Immunohistochemistry may be useful to differentiate BCC and SC, with SC being positive for epithelial antigen membrane (EMA), chorioallantoic membrane 5.2 (CAM5.2) (low molecular-weight keratin) and BRST-1 (marker for breast carcinoma), while BCC is typically negative for EMA and BRST-1. BCC, including BCC with adnexal or sebaceous differentiation, stains positively for epithelial cell adhesion molecule (Ber-EP4) [2]. The risk of metastasis for SC is highest in periocular lesions and ranges from 17% to 28% and typically spreads first to the regional lymph nodes [1]. Surgical treatment modalities include standard excision with 5- to 6- mm margins and Mohs micrographic surgery, with recurrence rates of 39.6% and 15.7%, respectively [1]. Patients should be monitored closely thereafter because of the risk for tumor metastasis and recurrence.

Collision tumors are two or more neoplasms of distinct cell populations occurring at the same cutaneous site [5]. The mechanism for the development of collision tumors is unknown. The most common accepted theory is explained by neoplastic heterogenicity. This theory hypothesizes that collision tumors result from two different clones of neoplastic cells by chance alone. Other theories include field cancerization, stem cell cancerization, and epithelial or stromal changes induced by the first neoplasm stimulates development of the second neoplasm [5].

## Conclusions

We report the second case of collision of BCC and SC and use this case to emphasize the importance of verifying biopsy pathology during Mohs surgery when frozen section evaluation reveals disparate findings to the originally biopsied tumor. Follow-up with permanent sections and immunohistochemistry may be necessary. We advise caution to detect mimickers of BCC that warrant greater clinical workup. Given the frequent periocular location of SC and the superior cure rate, we recommend Mohs micrographic surgery for SC.
